# Supramolecular Dimerization in a Polymer Melt from Small-Angle X-ray Scattering and Rheology: A Miscible Model System

**DOI:** 10.3390/polym12040880

**Published:** 2020-04-10

**Authors:** Mariapaola Staropoli, Margarita Kruteva, Jürgen Allgaier, Andreas Wischnewski, Wim Pyckhout-Hintzen

**Affiliations:** 1 Forschungszentrum Jülich, D-52425 Jülich, Germany; m.kruteva@fz-juelich.de (M.K.); j.allgaier@fz-juelich.de (J.A.); a.wischnewski@fz-juelich.de (A.W.); w.pyckhout@fz-juelich.de (W.P.-H.); 2 Luxembourg Institute of Science and Technology, L-4422 Belvaux, Luxembourg

**Keywords:** SAXS, rheology, heterocomplementary association, thymine, diamino-triazine, melt, TTS, hydrogen bonding, poly(butylene oxide)

## Abstract

We present a structural and dynamic study on the simplest supramolecular hetero-association, recently investigated by the authors to prepare architectural homogeneous structures in the melt state, based on the bio-inspired hydrogen-bonding of thymine/diaminotriazine (thy–DAT) base-pairs. In the combination with an amorphous low T_g_ poly(butylene oxide) (PBO), no micellar structures are formed, which is expected for nonpolar polymers because of noncompatibility with the highly polar supramolecular groups. Instead, a clear polymer-like transient architecture is retrieved. This makes the heterocomplementary thy–DAT association an ideal candidate for further exploitation of the hydrogen-bonding ability in the bulk for self-healing purposes, damage management in rubbers or even the development of easily processable branched polymers with built-in plasticizer. In the present work, we investigate the temperature range from T_g_ + 20 °C to T_g_ + 150 °C of an oligomeric PBO using small-angle X-ray scattering (SAXS) and linear rheology on the pure thy and pure DAT monofunctionals and on an equimolar mixture of thy/DAT oligomers. The linear rheology performed at low temperature is found to correspond to fully closed-state dimeric configurations. At intermediate temperatures, SAXS probes the equilibrium between open and closed states of the thy–DAT mixtures. The temperature-dependent association constant in the full range between open and closed H-bonds and an enhancement of the monomeric friction coefficient due to the groups is obtained. The thy–DAT association in the melt is more stable than the DAT–DAT, whereas the thy–thy association seems to involve additional long-lived interactions.

## 1. Introduction

Biological, ‘living’ materials have shown to be the most advanced functional systems, and their efficiency in complexity cannot be easily imitated artificially. They differ from ‘dead’ synthetic materials that we know from everyday life at least in their considerable ability to self-repair. Natural materials continuously and repeatably adapt. In consequence, they are autoresponsive to damage and suitable for prolonged use [1,2,3,4,5,6]. Synthetic conventional covalently crosslinked rubbers, on the other hand, are used in the form of seals or joints between different materials. During use they are subjected to fatigue, impact, abrasion and wear that cause the formation of minuscule fractures leading to further larger-scale unrepairable failures and to a limited lifetime. Therefore, transferring self-repair concepts from living matter to synthetic elastomers would represent a great benefit for extending the service intervals of rubber-based components or even for reaching new advanced materials [7,8,9,10,11,12,13]. Designing such novel materials for the future is an interdisciplinary process and requires the joint efforts of microscopic and macroscopic studies of the fundamental underlying principles [14]. Only then can knowledge-based transfer between different levels of complexity that distinguish nature and well-defined molecular–synthetic constructions be ensured.

However, the so-called self-repair of a material ending up with a final state having identical predamage properties is an almost unreachable aim. On the other hand, the introduction of supramolecular mechanisms in materials that are subjected to strong external constraints, reducing overstresses or overstrains at sensible positions, would be a viable alternative stabilization mechanism [9,15,16]. These functions are closely related to self-healing. Hydrogen-bonding interactions, among others, are highly reversible, and their binding is controlled by equilibrium association constants that depend on the embedding environment, the temperature and on the multiplicity of the established H…H bonds in the complex. Very often, however, the polarity of the H-bonding groups causes a natural energetic incompatibility between the groups and the polymer to which they are bound [14,17,18]. They would tend to microphase separation or micellarization in the melt and introduce new time dependencies in their properties that are linked to the morphology and structure of hydrogen-bonding nanodomains, rather than to the H-bond itself. Many examples of such randomly or terminally functionalized polymers with hydrogen-bonding interactions in solution and bulk state can be found in the literature [11,13,19,20,21,22,23,24,25,26,27]. In the present context, ionomers also structurally resemble the phase separation [28,29,30]. Likewise, metallo-intermediated supramolecular associations which tend to form homogeneous systems are known to behave in a similar way as well [31,32,33]. Their use is, however, limited related to the mixing of the components. The dynamics of supramolecular polymers in melt state was concisely reviewed [17]. In the bulk, the density of H-bonding groups is much higher than in solution, and therefore the chemical environment of the groups determines the group interactions in a decisive way.

Recently, Kruteva et al. identified a suitable model system for advanced bulk applications. It ideally combined electronic and thermodynamic properties of the active H-bonding entities and the polymeric backbone [34]. The pronounced compatibility of the matrix polymer and the functional groups is a significant aspect, as it may allow a further step to arrive at bulk materials with better and increased damage resistance [9]. Thymine (thy) and diaminotriazine (DAT) are structurally very similar to the base-pair thymine/guanine and are available in reasonable amounts [26]. They can be relatively easy implanted onto polymeric backbones like poly(ethylene oxide) (PEO) or generally poly(alkylene oxides) like poly(propylene oxide) (PPO) or poly(butylene oxide) (PBO). The association of thy and DAT groups in functionalized polymers like moderately polar PPO [22,23,35] and strongly apolar polyisobutylene (PIB) [8,36,37] has been extensively studied. For both systems, a microphase separation was reported. In combination with PEO and with equimolar amounts of difunctional unentangled oligomers, labeled by hydrogen and deuterium, small-angle neutron scattering (SANS) experiments in the melt state could identify uniquely linear –(HD)_n_– multiblock copolymers similar to polycondensation in the bulk [34]. The length of the dynamic chains was observed to be polydisperse and T-dependent. The results were corroborated by viscosity and pulsed-field gradient nuclear magnetic resonance (PFG-NMR) diffusion experiments, each delivering its moment of the molecular weight distribution. Very similar Hildebrandt solubility parameters that qualify miscibility were derived for both the groups and the poly(ethylene oxidic) backbone [38,39]. The Flory–Huggins parameter *χ* between thy/DAT and PEO derived from them was therefore negligible. In a more recent work, the apparently strongly preferred heterocomplementary thy–DAT association was applied to the formation of transient comb polymers based on PBO likewise directly in the bulk [40,41]. The study was highlighted in [17]. By a combination of SANS, rheology and broad-band dielectric spectroscopy, the mean lifetimes of the H-bonds in the bulk could be determined as being of the order of 1 s at −25 °C. From this result, a frequency of opening and closing on the order of ~100 Hz at +25 °C was estimated. Furthermore, the dynamics of the supramolecular polymer were found to be in very good agreement with those of the equivalent permanent covalent comb polymer with arms linked to the same statistical places where thy-units were built in [40]. This correlation supported the potential use of this supramolecular branched system for processing applications where branching and simultaneous plasticizing are important.

An increased compatibility of thy and DAT was also achieved in the literature with poly(butyl acrylate) (PBA) as the matrix [19]. However, the strong rise in the T_g_ by roughly 30 °C upon functionalization of the PBA polymer may be an indication for strong local effects as well, and it contrasts with the observations for the present PBO polymer [42]. In addition, PBO is a particularly interesting intermediate polar polymer because of its low T_g_ (−65 °C) and an entanglement molecular weight around 8000 g/mol. Both are very similar to the apolar polyisoprene (PI) or natural rubber (NR) elastomers [42]. PBA, though with comparably low T_g_, on the other hand possesses an entanglement molecular weight of about 30,000 g/mol and does not show valuable elastomeric properties on its own.

In the present work, we investigate the simplest mixtures of identical monofunctionalized short PBO chains to reveal the stability and nature of the thy/DAT H–H bond directly in the bulk. They will lead to homo-association and to hetero-association in equimolar thy/DAT compounds, characterized by the equilibrium dimerization constant $K_{eq}=\left[AB\right]/\left[A\right]\left[B\right]$ following A+B⇌AB [5,8,15]. A temperature range from T_g_ + 20 °C to T_g_ + 150 °C using linear rheology and small-angle X-ray scattering (SAXS) will be covered. Whereas from the former work [40], the time scale of bonding of ~1 s could be already estimated at −25 °C, the actual temperature dependence of the equilibrium constant K_eq_ (T) could not yet be accessed accurately. Therefore, this manuscript accesses the temperature-dependent structure and the dynamics in the bonded state directly instead of inferring them from macroscopic techniques only. The work attempts to provide a firm base for future work under mechanical or oscillatory deformation with medium-to-large amplitude as in real operation cases, thereby leading to a control of dissipation mechanisms.

## 2. Materials and Methods

### 2.1. Synthesis and Preparation of Mixtures

All poly(butylene oxide) (PBO) polymers were prepared by anionic ring-opening polymerization with subsequent chemical modification. The specific synthetic details and characterization by size-exclusion chromatography (SEC) (Polymer Laboratories, PL 220, Salop, UK) have been published elsewhere [42] and are repeated in short in Table 1. All samples were stored at −20 °C before use. Table 1 lists the polymer codes used in this work. Due to side reactions during the synthesis, PBO-5k-thy contained 1% covalently coupled dimeric product, and the functionalization degree with thy was 97%. For PBO-5k-DAT, 5% coupled dimeric product appeared, and at least 90% of the end-groups were capped with DAT units. For all systems, ^1^H-NMR spectroscopy (Bruker, Avance III 600 MHz, Karlsruhe, Germany) was used for the determination of the degree of functionalization. The equimolar mixture of the thy- and DAT-polymers was prepared in pentane. The solvent was removed under high vacuum for 4 days. The polymer melts were used directly after removing the solvent to avoid water contamination. Glass transition temperatures were determined from 10 °C/min runs.

### 2.2. Linear Rheology

Linear rheology experiments in oscillatory shear were carried out on a strain-controlled ARES (advanced rheometric expansion system) (Rheometrics Sci Ltd., Piscataway, NJ, USA) rheometer equipped with a 2K-FRTN1 (force rebalance transducer, normal force) transducer in the parallel plate geometry. The diameter of the plates was either 8 or 25 mm, and the sample gap was about 1 mm. Isothermal frequency sweeps were performed in the range between 0.1 < ω < 100 rad/s. A temperature range between −50 < T < +10 °C with ΔT = 10 °C under a nitrogen blanket was used, and strain amplitudes in the linear regime of the polymers were ensured. Samples were equilibrated between the plates for 15 min. Master curves were constructed by a two-dimensional time–temperature superposition (TTS) shifting procedure using the Orchestrator software (TA Instruments) at a reference temperature T0 of −25 °C for each sample. The horizontal shift factor a_T_ varied strongly with temperature, whereas the vertical b_T_ shift factor was well around 1. A Williams–Landel–Ferry (WLF) function was fitted for the investigated temperature range. The construction of the master curves followed perfect WLF behavior, indicating simple thermo-rheological behavior in the covered time and temperature range.

### 2.3. Small-Angle X-ray Scattering (SAXS)

SAXS experiments were conducted at the GALAXI diffractometer based in the Jülich Centre for Neutron Science-2 Institute (JCNS-2) at Forschungsszentrum Juelich, Germany [43]. The X-ray source utilizes a liquid metal jet target of a GaInSn alloy as the anode to which 70 keV electrons are sent. The resulting X-rays are monochromatized to allow only Ga K-α radiation of E = 9.243 keV photon energy to pass to obtain a wavelength λ = 1.34 Å. Two four-segment slits which are separated by 4 m distance collimate the beam and confine the size to about 0.7 × 0.7 mm^2^. A third slit reduces the scattering from the edges of the second one. A sample-to-detector distance of 80 cm, calibrated using Bragg reflections from silver behenate resulting in a q-range of 0.01–0.7 A^−1^ was used. Absolute intensities in inverse centimeters were obtained by the calibration with a secondary standard consisting of a hexafluoro-ethylene-propylene copolymer (Dupont). All samples were sealed in borosilicate capillaries of 2 mm nominal inner diameter and placed in the vacuum chamber. Standard corrections for cell scattering and detector efficiency were performed. The temperature was varied between +15 to +75 °C with an accuracy of about 1 °C using a thermostat.

## 3. Structural Model of Association

Monofunctionalized thy– and DAT– PBO oligomers are structural analogs to strongly asymmetric AB block copolymers [22,44,45,46]. For strongly incompatible blocks, the mixing of both components is enthalpically unfavorable, and typical microphase-separated domains can be found. Such an incompatibility of A and B blocks is reflected in a nonzero Flory–Huggins interaction *χ* parameter [44,46]. It determines particle-like or polymer-like behavior in the phase diagram. This interaction can be estimated—at least roughly—using an empirical approach based on the so-called solubility parameter δ [38,39]. *χ* is then a simple function of the squared difference of the solubility parameters of both components. A nonzero value for latter translates into a nonzero *χ* parameter. The absence of it or the size of a measurable *χ* interaction parameter between A and B moieties is typically obtained from SAXS or SANS experiments. The estimation was good in the case of PEO [34], and we will assume further that it will be approximately correct also for PBO.

Based on the experimental miscibility of thy/DAT and PBO deduced from the former studies [40,41,42], we propose in the following an N-star-diblock copolymer approach, which should be general to capture any signature of supramolecular star-like aggregates with a H-bonding core and with N arms attached to it. The calculation follows the same routes as in [41,47] and is therefore not detailed further here. It is based on a general random phase approach (RPA) for scattering [46,48] and is applicable to almost any particular architecture. The formed N-armed star then is composed of AB-diblock copolymer arms and summarized as (AB)_N_. N is the star functionality. If (ideal) dimerization with N = 2 takes place, a linear triblock structure of the type AB–BA is formed. If N = 1, the unimer diblock AB. For N > 2, a Gaussian star diblock copolymer (AB)_N_ results. The RPA structure factors for each are very similar.

The partial structure factors of the blocks are written as:

(1) $$P_{BB}=N\left(N-1\right)H^{2}\left(n_{B}\right)+NJ\left(n_{B}\right)$$

(2) $$P_{AA}=N\left(N-1\right)H^{2}\left(n_{A}\right)G\left(2n_{B}\right)+NJ\left(n_{A}\right)$$

(3) $$P_{AB}=NH\left(n_{A}\right)H\left(n_{B}\right)+N\left(N-1\right)H\left(n_{A}\right)H\left(n_{B}\right)G\left(n_{B}\right)$$

where B is the supramolecular central core-forming block, i.e., the tentative or possible phase-separating H-bonding groups in our approach. Intra- and interblock correlations are considered. The functions J, H and G are defined as:

(4) $$J\left(n\right)=\frac{2}{{\left(Xn\right)}^{4}}\left(exp\left(-{\left(Xn\right)}^{2}\right)-1+{\left(Xn\right)}^{2}\right)$$

(5) $$H\left(n\right)=\frac{1-exp\left(-Xn\right)}{{\left(Xn\right)}^{2}}$$

(6) $$G\left(n\right)=exp\left(-{\left(Xn\right)}^{2}\right)$$

where $X={\left({ql}_{st}\right)}^{2}/6$. $l_{st}$ is the statistical segment length per monomer and q is the scattering vector defined as $q=\frac{4\pi }{\lambda }sin\frac{\theta }{2}$. J is the well-known Debye function [38].

The structure factors are defined as:

(7) $$S_{AA}=\phi_{A}v_{0}{Nn}_{A}P_{AA}$$

(8) $$S_{BB}=\phi_{B}v_{0}{Nn}_{B}P_{BB}$$

(9) $$S_{AB}=\sqrt{\phi_{A}v_{0}{Nn}_{A}\phi_{B}v_{0}{Nn}_{B}P_{AA}P_{BB}}$$

Here, n_A_ and n_B_ denote the number of monomers in blocks A and B, respectively; $\phi_{A}$ and $\phi_{B}$ denote the volume fractions of blocks A and B, respectively. V_0_ is the monomer volume. For the interacting system, the RPA structure factor is as follows:

(10) $$S_{RPA}=\frac{S_{AA}S_{BB}-S_{AB}^{2}}{\left(S_{AA}+S_{BB}+2S_{AB}\right)-2\frac{\chi }{v_{0}}\left(S_{AA}S_{BB}-S_{AB}^{2}\right)}$$

We have omitted everywhere the q-dependence of the structure factors. *χ* refers to the affinity of the functional groups with the polymer backbone to which they are attached.

Some comments regarding the sensitivity of calculation are important here: For the thy and DAT groups an average solubility parameter δ ~24 is derived [27,34]. Referring to [34], the estimated δ parameter for PEO is 20.8, i.e., lower than the experimental literature value situated between 22 and 25 by roughly 2–3 MPa^1/2^ [39]. Nevertheless, from SANS, the mixture was found ideal with negligible Flory–Huggins parameter. We conclude therefore that for differences Δδ ~2–3 between experimental and the empirically estimated values from the group contribution approach, no or only a small *χ* parameter is to be expected in PEO-like systems with similar interactions. In other words, the estimated difference then leads to an offset for *χ* to be ~0.004. We will take this into account also for PBO and correct the estimated *χ* for this very same offset. For the present PBO case, the estimated δ value is 19 i.e., even smaller than for PEO [38,39]. Due to the similar chemical structure, however, and taking in account the same empirical offset, a final *χ* of ~0.008 would remain. With n_A_ ~70, *χ*n_A_ is 0.57; for this asymmetric short diblock with $\phi_{B}\leq 0.04,$ no microphase separation is expected.

In this SAXS study, for experimental reasons, the temperature range was limited to 15 °C up to 75 °C. As the supramolecular association is strongly temperature-dependent, simple binary mixtures of N-armed stars in equilibrium with unimeric diblocks have to be considered. No new correlations between A and B blocks in the unimer/N-mer mixture appear. The average aggregation number is determined from fits to the SAXS curves.

The SAXS experiments should address specific points. First, for the initial aim of exploiting the hetero-association for well-defined controllable interactions and well-defined properties in dense dual networks, the question of the stability of the thy–DAT bonds in the melt state at ambient temperatures is important [19]. In dual networks, the complexation is almost entirely restricted to the elastic mesh volume of the covalent network [9,49]. No diffusion over longer distances will be allowed, and the same partners are found. This contrasts strongly with the hydro- or organo-gel systems where the mobility is high due to the presence of solvent molecules [50,51]. Material is transported over longer distances and a strong partner exchange is likely to occur. The strongest bond in both bulk and solution is thy–DAT, but the order stability of thy–thy and DAT–DAT association is easily inversed [19]. Secondly, the question of the competitive homo-association of thy and DAT groups still stands. In the PEO case of [34], the probability for pure thy–thy or DAT–DAT homo-complexes was estimated to be roughly 1:3 when they are compared to the thy–DAT hetero-association.

**Figure 1:**
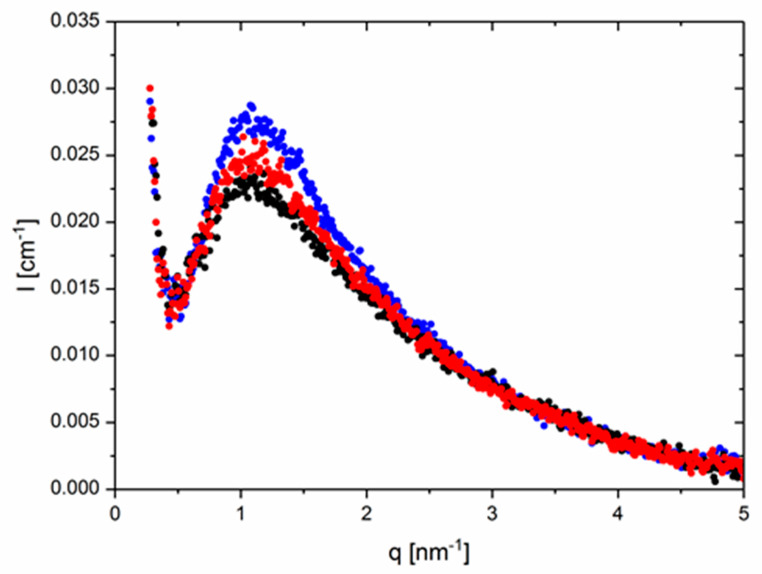
SAXS intensities of the thymine/diaminotriazine (thy–DAT) mixture, I (in cm^−1^) versus q (in nm^−1^). Here, an experimental background of the corresponding pristine PBO-5k obtained from superposition of the curves at q of ~8 nm^−1^ is subtracted. Only the relevant part of I(q) is shown. A parasitic scattering contribution following a q^−4^ dependence from void scattering is visible in the lowest q-region.

Figure 1 presents absolute SAXS intensity patterns of an equimolar monofunctional thy–DAT mixture as a function of temperature. The contrast is given by the electron density difference of the PBO backbone vs. the functional groups. The data were corrected for background scattering. The contribution of the parent PBO-5k was subtracted weighted with its volume fraction in the supramolecular cases. The intensities corrected as such indicate a clear block copolymer-like RPA correlation hole peak at q^*^ roughly ~1 nm^−1^ [46]. This corresponds approximately to a distance of 2π nm which is approximately the end-to-end distance of a gaussian chain with M_w_ = 5000 g/mol and 70 PBO monomers [52]. The peak height decreases with temperature possibly as the consequence of χ being inversely dependent on T and/or the deaggregation of the supramolecular chain. Importantly, in the intermediate q-range, a clear polymer behavior with a q^−2^ decay characteristic for the random walk structure is seen. This is in strong contrast to the usually found particle-like q^−4^ surface scattering of domains for the same groups but in a different matrix [36,37]. In the lowest q-range, a parasitic strong decay of the intensity with also approximately q^−4^ is also evident. This is assigned to electron-deficient voids or dust in the glass-sealed samples. It covers possible forward scattering intensity from fluctuations in the composition. Since the fraction of groups in the unimer is the same as in the coupled species, this mixing contribution is most probably absent or very low [46]. In the highest q-range for q > 3 nm^−1^, finally the q-dependent, increasing background, i.e., the left wing of the amorphous halo centered around a q value of ~10–15 nm^−1^ on top of the incompressibility scattering of PBO, is the main contributor. In summary, already these observations clearly prove the absence of typical particle-like scattering and hard-sphere interaction contributions and indicate no or only small incompatibilities of the groups with the polymer matrix. No phase-separation of groups is observed as in virtually all other known supramolecular (e.g., [36,37]) or ionomeric systems [28,29,30]. For stability purposes in the fitting process, however, the respective backgrounds will be modeled by a polynomial function up to q^4^ following a procedure suggested by Vonk [53], rather than using the experimental (q-dependent) pristine PBO-5k which induces larger or uncontrolled uncertainties at higher q.

**Figure 2:**
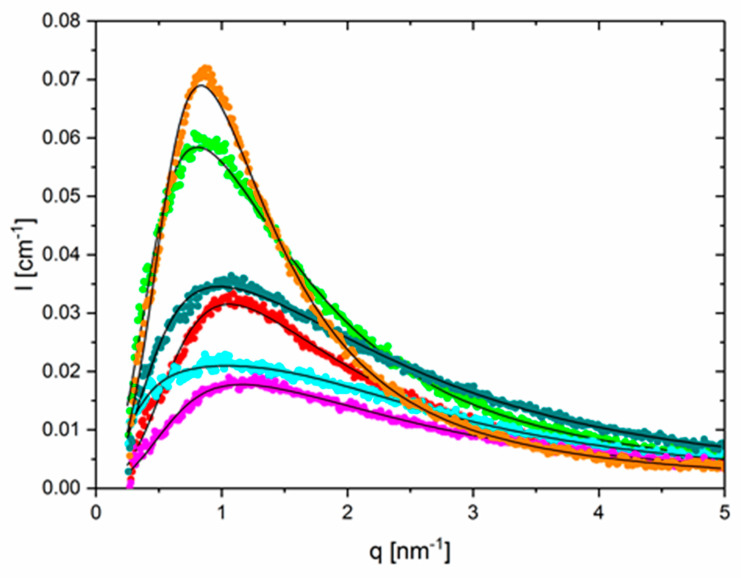
SAXS data at 15 and 75 °C for the three mixtures after correction for the q-dependent background. Parameters are discussed in the text.

Figure 2 summarizes the scattering intensity at 15 and 75 °C for the three combinations: thy–thy, DAT–DAT and thy–DAT. Here, the degree of homo-association between the groups of thy and DAT can be fitted and compared to the hetero thy–DAT case using the former RPA-based structure factor. The parasitic scattering at low q and background were removed. The q-independent fitted background contribution at q = 0 was only 0.006 cm^−1^, i.e., ~25% higher than the approximately computed isothermal incompressibility scattering of PBO on the basis of group contributions [39]. The deviation is within the uncertainties of the computation and could also be associated to the nonideal composition fluctuations and inaccurate thicknesses of the capillaries.

**Figure 3:**
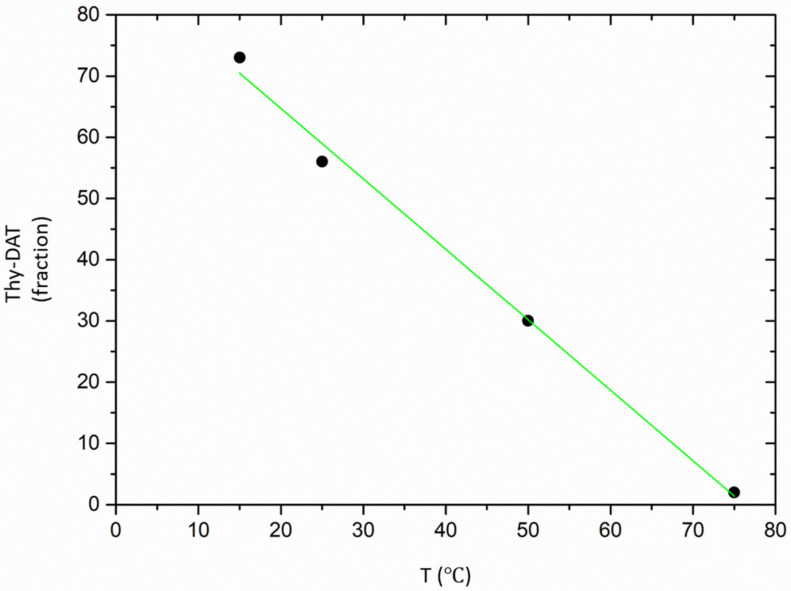
Dimer fraction in percent of thy–DAT extrapolated out of the 15–75 °C range towards −25 °C. Within experimental error, the dimerization is complete at the rheological reference temperature.

Figure 2 shows the most pronounced peak for thymine and the shallowest one for DAT, whereas the thy–DAT mixture is located in between. The fit curves to our suggested polymer model are shown as solid lines. Here, the monomer number of the arms n_A_ was kept fixed to 69, i.e., as obtained from SEC, and l_st_ was allowed to vary [42]. The supramolecular groups were computationally replaced by three additional effective PBO monomers n_B_, on average with the same statistical segment length, to simulate at best the end-to-end distance of the H-bonded complex. In addition, we fitted the average functionality N and χ. If we assume that the mixture only consists of N-associated stars and unimers, the respective fraction can be obtained. From Figure 2, a clear homo-association is found for thy–thy. This differs already from the PBA case, where no thy–thy association could be traced [19]. The initial average functionality at 15 °C was N_thy_ = (2.9 ± 0.3) and could comply to a star-like trimer (or similar) present for 95%. N_thy_ reduces to (2.1 ± 0.1), i.e., a linear dimer for 60% at T = 50 °C. Finally, at 75 °C the average functionality is (1.7 ± 0.2) and corresponds to 85% of dimer in equilibrium with the unimers. We note that thymine is known to form π–π stacks [26]. Possibly the star-like geometry only parametrizes the tendency of some stacking interactions. The opposite is found for the DAT–DAT combination. N_DAT_ = (1.5 ± 0.2) already at 15 °C, meaning 52% dimers only, and from 25 °C on ~100% unimers are found. Again, for PBA, the DAT–DAT interaction is about as strong as the thy–DAT. For the most interesting thy–DAT equimolar mixture in PBO, a different behavior is observed. At 15 °C, the association degree N_thy–DAT_ = (1.7 ± 0.1) corresponds to 73% of dimer and ends up at the unimer level with N_thy–DAT_ = (1.0 ± 0.1) with <2% remaining dimer at 75 °C. In Table 2, the respective parameters are summarized, and the constant of dimerization K_eq_ is estimated using the mass balance of unimers and dimers. Numerically they are much lower than the dimerization in apolar solvent. However, a good correspondence with our former work on PEO exists. In that case [34], the K_eq_ at which the supramolecular chain only consists anymore of roughly two unimers, a comparable size of K_eq_ ~10 L/mol is found, irrespective of the temperature of observation. We attribute this to the similar backbone chemistry of PEO and PBO. Furthermore, a tentative linear extrapolation of the dimer fraction as in Figure 3 from this T-range to the reference temperature of rheology of −25 °C—see below—leads to 105% ± 2%, thus in agreement with a fully dimerized state.

The derived χ-parameters were 0.016, 0.012 and 0.019 with error bars of the order of 20–30% for the thy, DAT and the thy–DAT combinations, respectively. These compare within factors of 2 with the estimated ones from the mean-field-like solubility parameter approach [38,39]. Due to the rather small T-range in this investigation, no reliable dependence on T could be extracted.

The basic and average segment length for thy–DAT PBO, l_st_ = 0.72 nm, is in fair agreement with the reference [41] using SANS. Its size is somewhat larger here due to the approximately 4-fold higher contribution of supramolecular groups in the ratio 3:70 (n_B_:n_A_), whereas it was about 10:1000 monomers in the transient thy/DAT comb case of [41]. The thy–thy association, on the other hand, leads to an larger or stiffer average step size of about ~0.9 nm. On the contrary, DAT–DAT leads to an even smaller statistical segment length of ~0.65 nm. This finding indicates that the size of the included supramolecular group matters in each of them. DAT is more compact than thy. The effective statistical segment step size is a necessary parameter of the model. The observed stretching-out of the arm in the alleged three-armed thy-star can be a consequence of the nonplanarity of the H-bonds formed and an increase in the steric hinderance [26]. In the other two combinations, the H-bonding is more co-planar. We also note that thy–thy and DAT–DAT only form binary H…H associations, whereas a triple H-bond is formed between thy and DAT entities. Table 2 summarizes the temperature-dependent equilibrium association constants K_eq_ of dimerization and respective fraction of closed bonds for the thy–DAT case.

As a first conclusion, the existence of associated end groups results in polymer-chain-like aggregates which behave Gaussian-like and show random-walk behavior. No indication for a microphase separation could be found, in agreement with former experiments that had led to ideal comb-like architectures. The equilibrium constant of the dimerization is a sensitive function of temperature and limits the preferred applicability of the hetero-association for future dual networks with controlled properties towards temperatures below RT. The disparity with PBA in the order of homo-association consolidates the importance of the detailed chemical environment of the supramolecular groups.

## 4. Linear Rheology

The former structural SAXS study of the thy–DAT association already indicated from extrapolation that the degree of H-bonding would be virtually complete at the reference temperature T0 of −25 °C, at which all former work on PBO was more or less accidently mastered to [40,41,42]. The dimerization should have a clear effect on the dynamics of the hydrogen-bonded complex chains and the relaxation time spectrum.

**Figure 4:**
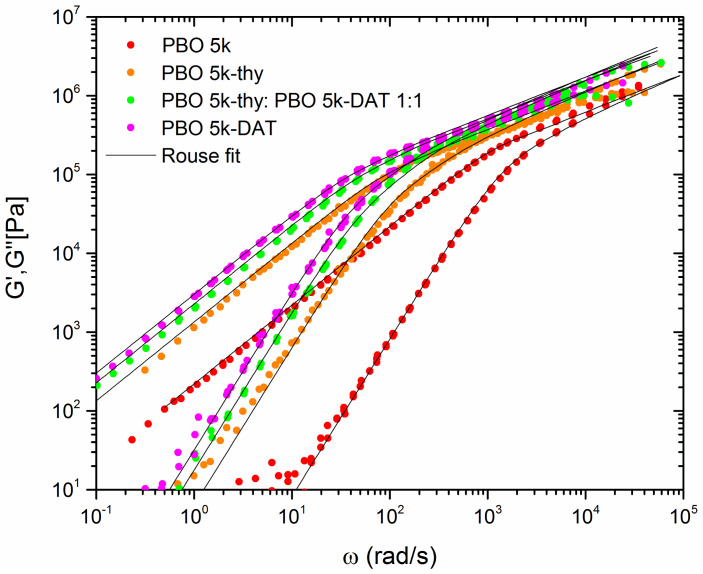
Storage G′(*ω*) and loss modulus G″(*ω*) master curves, reduced to T0 = −25 °C of unfunctionalized and functionalized PBO-5k compounds. Fits to the Rouse model are included.

Figure 4 presents the respective storage and loss moduli of the monofunctionalized and pure PBO oligomers. The DAT–DAT dimer is slower than the thy–thy associate and the hetero combination, which could be the result of the stronger interaction and stabilization in a planar arrangement of hydrogen bonds. The curves were shifted by means of the TTS procedure towards the reference temperature T0 and agree with a WLF behavior. This is corroborated by the analysis in [40,41] in the temperature range T < T0. There, deviations from the WLF form were observed only for T >> T0 as the result of the opening of H-bonds in mixtures of open and closed states. As the molecular time scales of the present PBO oligomers are well below the life time of the hydrogen bonds, the latter should occur as rigid, and no mixing of species is expected (see below). Thus, in this temperature range the mastered moduli show a perfect linear and fully associated chain behavior, as in the former studies in which side-branched PBO copolymers and blends were investigated. Visually, G′(*ω*) and G″(*ω*) show a perfect Rouse behavior with *ω*^1/2^ at high frequencies and the expected *ω*^2^ and *ω*^1^ at low frequencies, respectively. No signatures of longer relaxation times due to star-like configurations or other larger aggregates, stacks or micelles that would considerably broaden the relaxation time spectrum can be spotted. In the transition region, also no plateau is observed, typical for Rouse chains below or around the entanglement molecular weight.

However, a very strong upward shift in the characteristic times is clear from Figure 4. The parent PBO-5k and the functionalized ones are roughly separated in time by one order of magnitude. At the same time, the general Rouse properties are maintained, allowing to conclude that no large structural differences are responsible for the slowing-down of the functionalized systems. For the broadening of the spectrum, several factors are responsible [54]. First of all, the glass transition temperature of the functionalized oligomers is ~4 °C higher than that of PBO-5k [42], whereas that of the thy- and DAT-oligomers is comparable. The temperature gap between T0 and T_g_ while mastering to the reference temperature is therefore smaller for the thy- and DAT-modified oligomers. This leads to a slowing-down equivalent to a down-shift in the *ω*-axis compared to the parent PBO-5k. The shift in the time axis due to the slightly higher T_g_ can be judged with good accuracy from the ratio of the respective shift factors for the temperature difference. This ratio also applies to the monomeric friction coefficient and amounts to about 2.6 if the universal C1 and C2 parameters of the WLF function with C1 = 17.44 and C2 = 51.6 K are used. The monomeric friction coefficient is thus higher. Secondly, based on the former SAXS evaluations, the consequence of the thy and DAT groups associating with each other would obviously be a doubling of the chain length. Since the Rouse time scales like τ_R_ ~ n^2^, with n being the number of monomers in the chain, the time scale between the parent PBO-5k oligomer and the associated systems at iso-friction conditions should be extended by a factor of ideally 2^2^ = 4. Last but not least, an additional reason is a larger average monomeric friction coefficient due to the bulkier end groups. This would correlate with the experimental upshift of the T_g_ by about 4 °C and a shift in the relaxation time spectrum. Two of them can be corrected for. The total estimated shift in the frequency axis from these two effects would be 4 × 2.6 = 10.4. As we discuss below, the real shift is ~15, as illustrated in Figure 4. Every additional factor is therefore assigned to friction enhancement of the groups. We propose as a simple approximation that the effective monomeric friction coefficient in the supramolecular cases can be factorized as follows:

(11) $$\xi_{0}\left(T\right)=\xi_{0}^{PBO}\left(T\right)\times C_{T_{g}}\left(T\right)\times C_{f}$$

$C_{T_{g}}$(T) corrects for free volume and chain ends and is calculated easily from the ratio of the WLF dependencies of the a_T_ shift factors to their respective glass transition temperature. As a comparison, for oligomeric polybutadiene (PB) such an experimental factor is found roughly between 0.5 and 3, depending on the molecular weight [55].

$C_{f}$, on the other hand, denotes the correction to the monomeric friction coefficient due to the groups and is not supposed to be T-dependent. It cannot be estimated a priori. For thy and DAT groups, Kruteva et al. [34] estimated an increase of the friction $C_{f}$ by 1.14 per group in a PEO matrix. For a better quantification in our PBO case, however, especially because the polymer matrix itself may be a parameter, we will refer to a long sufficiently entangled PBO-38k polymer with M_w_ = 38,000 g/mol which we analyzed by means of the Likhtman–McLeish model [54]. Here, contour length fluctuations and constraint-release are accounted for analytically in the best possible way. From this, we extract the basic molecular time scales and can make predictions for those of the PBO-5k. The basic time step in the dynamics is the entanglement time $\tau_{e}$, which is defined as the Rouse time of a chain of mass M_e_ between two entanglements. We fitted $\tau_{e}\;$ to be (2.2 ± 0.1) × 10^−3^ s at −25 °C and the initial modulus G_e_ was 0.30 ± 0.02 MPa, corresponding to M_e_ = 7800 ± 600 g/mol. For Rouse dynamics, the total $\xi_{0}\left(T\right)$ and the Rouse time of the chain $\tau_{R}$ are directly related by

(12) $$\tau_{R}=\frac{\xi_{0}n^{2}l^{2}}{3\pi^{2}kT}$$

Substituting n by n_e_ = 111, i.e., the number of monomers in M_e_, the monomeric friction coefficient for pure unmodified PBO at −25 °C is found to be $\xi_{0}$ = 4.28 × 10^−8^ Ns/m. The corresponding Rouse time for a PBO-5k chain is estimated then for our oligomer with M_w_ = 0.62 M_e_ and yields (8.45 ± 0.38) × 10^−3^ s. The direct fit with the discrete Rouse model with

(13) $$G^{\times }\left(\omega \right)=\frac{\rho RT}{M}\overset{p=n}{\underset{p=1}{\sum }}\frac{{\left(\omega \tau_{p}\right)}^{2}+i\omega \tau_{p}}{1+{\left(\omega \tau_{p}\right)}^{2}}$$

to the dynamic moduli of the oligomer yields $\tau_{R}$ = (7.00 ± 0.02) × 10^−3^ s for the longest mode with *p* = 1 in sufficiently good agreement. Any difference could be due to either a maximum molecular weight discrepancy of 10% only, well within the accuracy of SEC, or most likely due to a combination of smaller uncertainties on the statistical segment length, the chain length and the monomeric friction coefficient. Table 3 summarizes the Rouse model fits to the different oligomers. Note that all times are considerably shorter than the H-bond life time of ~1 s at −25 °C, which was confirmed abundantly in the comb architecture by both rheology and dielectric spectroscopy without TTS application [40,41].

The fitted experimental Rouse times are presented in Table 3. We note that the experimental Rouse time of the equimolar mixture agrees with the average of the single components. This simplification allows us to consider the two groups, thy and DAT, as dynamically similar and averaged in the future. The dynamic shear moduli behave ideally as double-sized nonentangled Rouse chains with modified monomeric friction coefficient, as the corresponding fit curves prove unanimously. The new chain is then of the size 1.24 M_e_ and it is an experimental fact that for M < M_c_, Rouse theory should still apply. M_c_ is the critical molecular weight, being M_c_~2M_e_ at which the melt viscosity changes over from a M^1^ dependence into M^3.4^ due to the onset of tube constraints [54]. This internal consistency corroborates the complete dimerization of the end-group-functionalized oligomers at least at −25 °C. No contribution of unimers could be detected. The former trace of an approximately three-armed star-like configuration for the thy-functionalization from the SAXS experiment at 15 °C also apparently has no effect on the dynamic modulus. The slightly faster thy–PBO in its homo-associated state has the same span molecular weight as the others, obviously leading to a very similar qualitative response in rheology compared to that of a linear chain.

We restrict ourselves to the thy–DAT case for the interpretation of both correction factors $C_{T_{g}}\left(T\right)$ and $C_{f}$. The experimental ratio in Table 3 between the (longest) Rouse times of the thy–DAT mixture and PBO-5k at T0 = −25 °C is 15.7. The ratio would be 10.4 if only the dimerization and the temperature correction for the higher T_g_ are considered. So, coming back to our initial aim of obtaining $C_{f}$, the additional enhancement of the monomeric coefficient should be 1.51 for an average thy–DAT supramolecular complex or equivalently 1.25 for each average supramolecular group. This is comparable to the estimate from PFG-NMR results in PEO [34]. The further increase is possibly due to the contribution of the ethyl side branch in PBO.

From the quantitative molecular rheology fitting of the heterocomplementary thy–DAT association, it could be demonstrated that a full dimerization of all groups occurs at the reference temperature of −25 °C. Mixtures of dimers and unimers should be observed only at higher temperatures. A consistent monomeric friction enhancement for the groups was found, which is the same for both thy and DAT functional groups. No phase separation or micellarization whatsoever was observed in neither SAXS nor the dynamic moduli. The pure dimeric configurations guarantee very homogeneous mixtures. They are essential for e.g., damage management applications within dual networks at somewhat lower temperatures than ambient and are the best and promising requisites for controllable dissipation mechanisms.

## 5. Summary and Conclusions

In summary, it was shown from the pronounced q-dependence of the SAXS data for all samples that the bulk association of thy and DAT groups leads to a polymeric chain behavior, different from the usual observations of micellar particle- or hard-sphere-like assemblies. The strength of the interaction depends, however, on the different group combinations. Even some indication for an additional supramolecular interaction leading to some stacking configurations could be suspected for the thymine-modified PBO. We conclude that the supramolecular linear chain-like behavior is structurally driven by the compatibility of the groups with the chemistry of the polymeric PBO backbone, which plays a not yet fully understood role. The homo- and hetero-association of the groups was confirmed by the linear rheological behavior, and the existence of purely closed states at lower temperatures was disclosed. The SAXS analysis at higher-than-ambient temperatures could discriminate between associated closed and dissociated open structures. From their ratio, the dimerization constant of thy–DAT in the bulk as a function of temperature could be assigned. A good comparison with the related dimers of thy–DAT in the similar PEO at higher temperatures was even observed. With this knowledge and the temperature dependence of association, we have paved the way to incorporate the directional thy–DAT association into covalently crosslinked networks of long PBO chains which then should be responsive on the life time of the association in the temperature range of −25 < T < +25 °C.

## Figures and Tables

**Table 1:** Sample list and molecular weight characterization.

Sample Code	M_n_ (NMR) (g/mol)	M_w_/M_n_ (SEC) (g/mol)	T_g_ [°C] (±1 °C)
PBO-5k	4810	1.03	−68
PBO-5k-thy	4950	1.03	−63
PBO-5k-DAT	4870	1.06	−65
PBO-38K	38,400	1.02	−66

**Table 2:** Thermodynamic parameters derived from the structural model fitting of the thy–DAT equimolar mixture. The statistical segment length of the resulting associates varied between 0.72 < l_st_ < 0.79 nm in the considered T-range.

T (°C)	% Dimer thy–DAT	K_eq_(T) (L/mol)
15	73	9.5 ± 2.5
25	56	5.7 ± 2.3
50	30	1.2 ± 2.3
75	2	n.a.

**Table 3:** Rouse model fit parameters. For the functionalized derivatives, the chain length was doubled in the calculation.

Sample	τ_R_ (s) @ −25 °C
PBO-5k	(0.70 ± 0.02) × 10^−3^
PBO-5k-thy	(0.85 ± 0.02) × 10^−2^
Equimolar PBO-5k-Thy/PBO-5k-DAT	(0.11 ± 0.04) × 10^−1^
PBO-5k-DAT	(0.21 ± 0.05) × 10^−1^
